# Meta-analysis of the adjuvant treatment of lung cancer patients with Erchen Decoction

**DOI:** 10.1097/MD.0000000000045774

**Published:** 2025-10-31

**Authors:** Xueyan Lv, Dongmei Wang, Xiangni Zou

**Affiliations:** a Heilongjiang University of Chinese Medicine, Harbin, China; b First Affiliated Hospital of Heilongjiang University of Chinese Medicine, Harbin, China.

**Keywords:** analysis, Erchen Decoction, lung cancer patients, meta, traditional Chinese medicine

## Abstract

**Background:**

To systematically evaluate the effect of the adjuvant treatment of lung cancer patients with Erchen Decoction.

**Methods:**

Systematic electronic searches were conducted in multiple databases, including CNKI, Wanfang, VIP, EMBase, Scopus, Web of Science, PubMed, and the Cochrane Library. The objective was to retrieve all randomized controlled trials investigating the use of Erchen Decoction as an adjunct therapy for lung cancer patients. The search spanned from the inception of each database up to October 2024, and additional studies were identified by cross-checking the bibliographies of the included articles. Two independent reviewers performed study selection, data extraction, and methodological quality assessment. RevMan 5.3 software was utilized for meta-analysis, while the Cochrane system was applied to evaluate the evidence quality.

**Results:**

Eight studies were included, with a total of 550 study participants. They were randomized into experimental and control groups. The indicators included effective rate, CD3, CD4, Karnofsky performance status, forced expiratory volume in 1 second, etc. The differences between the 2 groups before treatment were not statistically significant (all *P* > .05). After treatment, the indicators of the experimental group were significantly better than those of the control group, and the differences were all statistically significant (*P* < .05).

**Conclusion:**

Erchen Decoction demonstrates a quicker amelioration of symptoms in lung cancer patients, thereby facilitating the decrease of multiple risk indices. Nevertheless, constrained by the scarcity of high-quality literature, future research should prioritize large-sample, double-blind, randomized trials to reinforce its theoretical foundation.

## 1. Introduction

Smoking is recognized as a major risk factor for lung cancer,^[1,2]^ and a lot of evidences suggest that approximately 80% to 90% of lung cancer cases are strongly associated with active or passive smoking.^[3]^ Unprocessed biomass fuels, asbestos, arsenic and radon, can also contribute to lung cancer incidence in certain parts of the world.^[4]^ Lung cancer is mainly characterized by respiratory symptoms, including cough, hemoptysis, chest pain and shortness of breath,^[5]^ and in recent years, it has been found that bronchial lung cancer patients’ peripheral blood cluster of differentiation 3 (CD3^+^) and cluster of differentiation 4 (CD4^+^) are significantly lower than those of the healthy population, which suggests that immune dysregulation is an important factor in the development of malignant tumors.^[6]^ Therapeutic options for lung cancer treatment include surgery, radiation therapy, chemotherapy, and targeted drug therapy.^[7]^ Erchen Decoction is a traditional Chinese medicine (TCM) formula. It is an expectorant with the effect of drying dampness and resolving phlegm.^[8]^ Erchen Decoction, which consists of Chen Pi, Pinellia, Poria and Licorice, has been widely used in the treatment of lung cancer patients in China for hundreds of years.^[9]^ In recent years, there has been a gradual emergence of randomized controlled trials (RCTs) on the application of Erchen Decoction as an adjuvant treatment for lung cancer. Er Chen Tang can improve cough by inhibiting the inflammatory response of the body, and its mechanism of action may be related to the mediation of the TGF-β/Smad signaling axis.^[10]^ This paper presents a systematic evaluation and meta-analysis of its efficacy.

## 2. Materials and methods

### 2.1. Data sources

#### 2.1.1. Literature inclusion criteria

① The study must be a RCT focusing on the adjunctive use of Erchen Decoction for treating lung cancer patients, with all essential components reported.
② The literature should detail the diagnostic methods and criteria for lung cancer patients.
③ The experimental and control groups must exhibit comparability in baseline characteristics.

#### 2.1.2. Exclusion criteria

① Studies with unclear origins of research participants.
② Literature with incomplete outcome data lacking reasonable explanations for data absence.
③ Duplicate publications across different databases.

### 2.2. Methodology

#### 2.2.1. Search strategy

Using “Lung Cancer,” “SCLC,” “NSCLC,” “Erchen Decoction,” “Erchen,” “Randomized Controlled Trial,” and “RCT” as search terms, a combined approach of MeSH terms and free-text words was employed. Comprehensive electronic searches were conducted in major academic databases, including CNKI, Wanfang, VIP, PubMed, EMBase, Scopus, the Cochrane Library, and Web of Science. The objective was to retrieve RCTs on Erchen Decoction as an adjunct therapy for lung cancer patients, supplemented by a manual search of bibliographies from included studies. The search period spanned from the database inception to October 2024. The search strategy is as follows: ((“Lung Neoplasms”[MeSH Terms] OR lung cancer OR lung neoplasm* OR “Pulmonary Neoplasms”[MeSH Terms]) AND ((“Erchen Decoction” OR “Erchen Tang” OR “Two-Cured Decoction” OR “Er Chen Tang” OR “Erchen Formula”) AND (treatment OR therapy OR intervene* OR effect* OR manage* OR clinical application))).

#### 2.2.2. Literature screening and data extraction

Two independent reviewers performed the initial screening process. They separately reviewed titles and abstracts, removed duplicates, excluded studies failing to meet inclusion criteria, and retrieved full-texts of eligible articles. Subsequently, the reviewers cross-checked inclusion decisions, conducted quality assessments, and consulted a third reviewer to resolve discrepancies. Data extraction covered publication date, inclusion and exclusion criteria, participant demographics of both groups, intervention details, and measured outcomes. For RCTs with incomplete long-term data, the original articles were scrutinized to identify reasons for data loss.

#### 2.2.3. Literature quality assessment

Based on the “Cochrane Collaboration’s Risk of Bias Assessment Criteria,” 2 reviewers independently evaluated the quality of included RCTs. The assessment covered randomization methods, concealment of allocation, blinding of participants and researchers, blinding of outcome assessment, completeness of outcome data, and selective reporting. The evaluation results are “low risk,” “high risk,” and “unclear.” If each document fully meets the evaluation content, the document quality is grade A; if it partially meets, the quality is grade B; if it does not meet at all, it is grade C, and grade C documents are excluded.

#### 2.2.4. Grade evidence grading evaluation

Use GRADE profiler 3.6 to evaluate the evidence quality, which is divided into: high, moderate, low, and very low levels. The evaluation content includes 5 aspects: risk of bias, inconsistency, indirectness, imprecision, and publication bias.

#### 2.2.5. Conclusion indicators

Effective rate; CD3^+^; CD4^+^; Karnofsky performance status (KPS); and forced expiratory volume in one second (FEV1).

### 2.3. Statistical analysis

Meta-analysis was performed using RevMan 5.3 software. Measurement data were evaluated by mean difference, and count data were evaluated by relative risk, and heterogeneity was determined by *I*^2^ test, *I*^2^ < 25% for no heterogeneity, 25% ≤ *I*^2^ < 50% for mild heterogeneity, all indicating that the difference was not statistically significant, and meta-analysis applied fixed effect model; 50% ≤ *I*^2^ < 75% for middle heterogeneity, *I*^2^ ≥ 75% for severe heterogeneity, all indicating statistically significant differences, meta-analysis applying a random-effects model, and if feasible, sensitivity analyses, and subgroup analyses should be performed to explain the source of heterogeneity. Meta-analysis applying a funnel plot to identify publication bias.

## 3. Results

### 3.1. Literature search results

**Figure 1.**
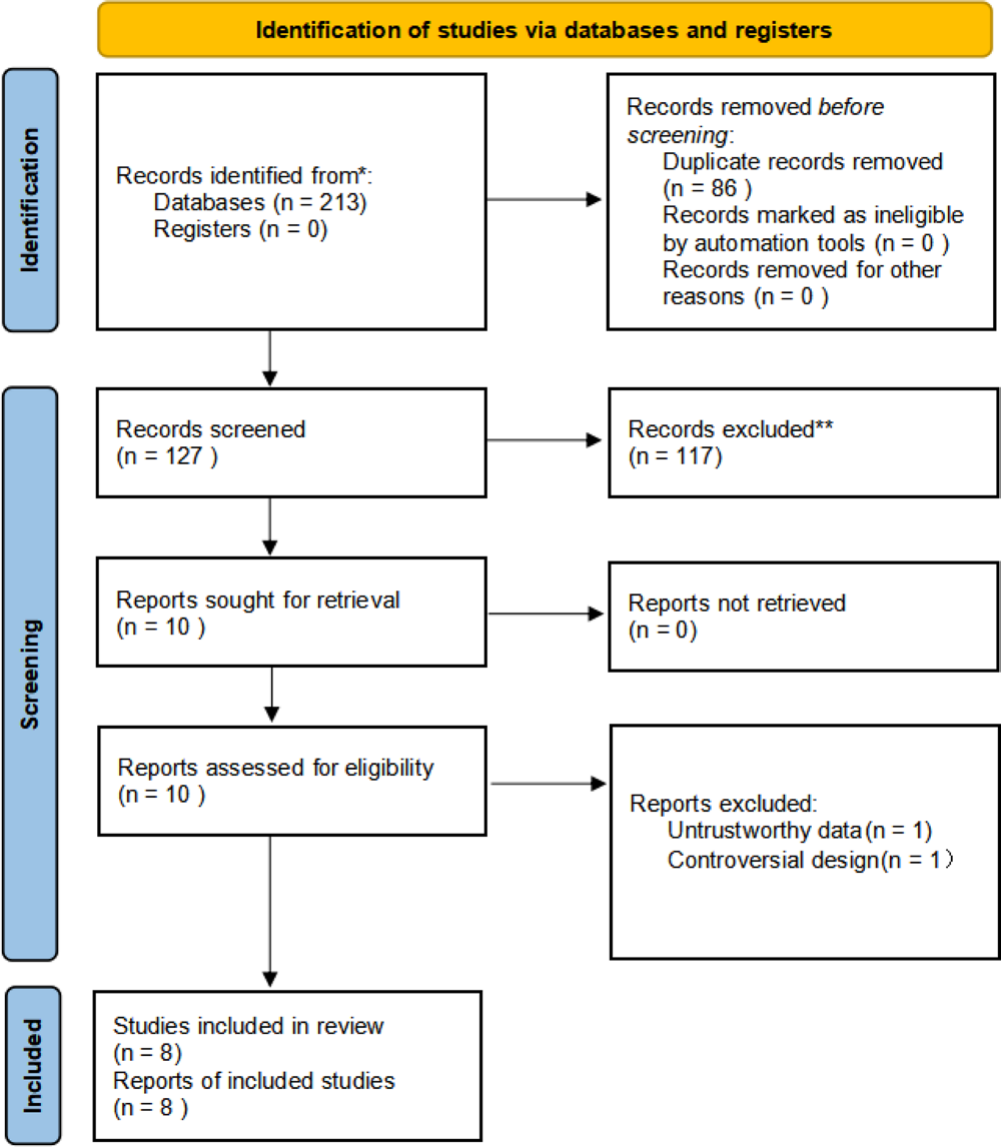
Literature screening process.

A total of 213 documents were initially examined, after excluding 86 duplicate articles, 127 articles remained. After careful reading of the abstracts, 117 non-RCT articles were excluded. After reading the full-texts, 2 low-quality articles were excluded. Finally, 8 articles were included in this study. There were a total of 550 patients, including 275 cases in the control group and 275 cases in the experimental group. The process of literature screening is shown in Figure 1, and the basic information of inclusion analysis is shown in Table 1.

**Table 1:** Basic characteristics of the 8 studies included in the meta-analysis.

First author	Year of publication	Integrate into number of examples		Age (yr)		Intervention		Outcome
		Test group	Control subjects	Test group	Control subjects	Test group	Control subjects	
Liang	2019	20	20	65.43 ± 5.21	65.25 ± 5.25	Lcotinib, 125 mg, po, tid + Erchen Decoction combined with GualouXiebaiBanxia Decoction, po, 1 does/d, course of treatment 14 d	Lcotinib, 125 mg, po, tid, course of treatment 14 d	①②③
Zhao	2017	30	30	70.85 ± 8.68	70.71 ± 9.96	Erchen Decoction, 100 mL, po, bid + symptomatic treatment + moxibustion + gemcitabine and cisplatin chemotherapy, course of treatment 84 d	Symptomatic treatment + gemcitabine and cisplatin chemotherapy, course of treatment 84 d	①
Li	2019	39	39	61. 9 ± 4. 2	62. 5 ± 4. 0	Symptomatic treatment + nedaplatin and gemcitabine chemotherapy + Erchen Decoction, po, 1 does/d, course of treatment 56 d	Symptomatic treatment + nedaplatin and gemcitabine chemotherapy, course of treatment 56 d	①②③④
Xie	2018	25	25	61.25 ± 5.74	62.21 ± 5.52	Symptomatic treatment + nedaplatin and gemcitabine chemotherapy + Erchen Decoction, po, 1 does/d, course of treatment 56 d	Symptomatic treatment + nedaplatin and gemcitabine chemotherapy, course of treatment 56 d	①②③
Gao	2022	40	40	0.30 ± 9.61	59.70 ± 10.94	Symptomatic treatment + compound methoxyphenamine capsules, 2 tab, po, tid + Erchen Decoction, 100 mL, po, bid + rehabilitation	Symptomatic treatment + compound methoxyphenamine capsules, 2 tab, po, tid	①⑤
Lei	2024	31	31	62.77 ± 5.61	63.14 ± 5.40	Jiawei Erchen Decoction, po, 1 does/d + paclitaxel and cisplatin chemotherapy, course of treatment 63 d	Paclitaxel and cisplatin chemotherapy, course of treatment 63 d	①④
Liu	2023	40	40	54.24 ± 6.98	54.31 ± 6.75	Compound methoxyphenamine capsules, 3 tab, po, tid + integrated breathing training + Erchen Decoction, 250 mL, po, bid + acupuncture, course of treatment 21 d	Compound methoxyphenamine capsules, 3 tab, po, tid + integrated breathing training, course of treatment 21 d	②③⑤
Che	2017	50	50	53.4 ± 8.3	53.4 ± 8.3	Nedaplatin and gemcitabine/gemcitabine and cisplatin chemotherapy + Erchen Decoction, 200 mL, po, bid, course of treatment 14 d	Nedaplatin and gemcitabine/gemcitabine and cisplatin chemotherapy, course of treatment 14 d	①④

① Effective rate; ② Cluster of differentiation 3 (CD3^+^); ③ Cluster of differentiation 4 (CD4^+^); ④ Karnofsky performance status (KPS); ⑤ Forced expiratory volume in 1 second (FEV1).

### 3.2. Literature quality evaluation

**Figure 2.**
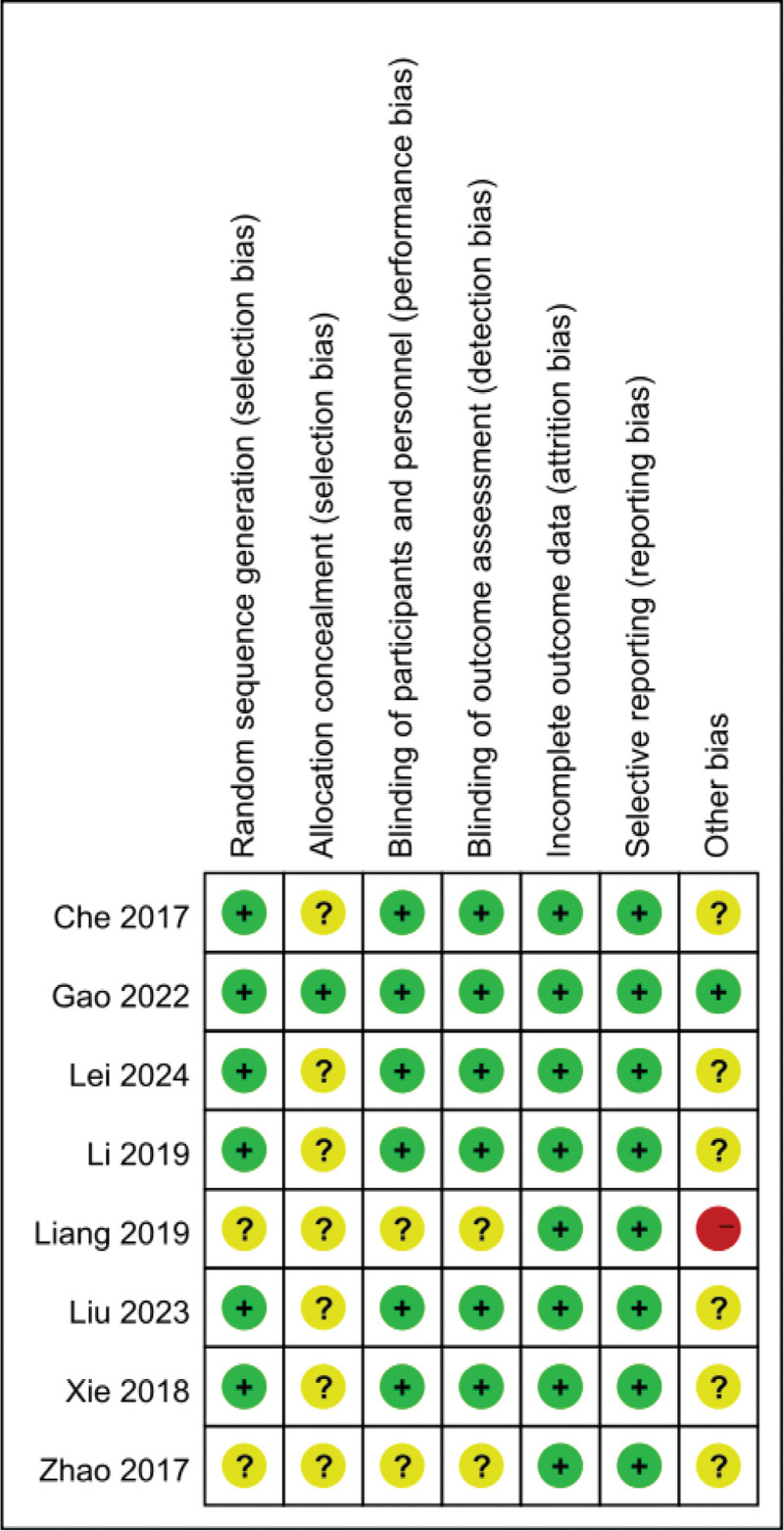
Risk of bias assessment for each study.

All the experimental designs included in the analysis are reasonable. Two of them do not mention random grouping, and 2 do not mention blinding, but the outcome indicators are all fully reported. The only shortcoming is that only 1 article mentions the allocation scheme masking. To sum up, the quality of evidence is of moderate quality. The quality of the 8 included literature^[11–18]^ was evaluated and the results of the quality evaluation are shown in Figure 2.

### 3.3. Meta-analysis results

#### 3.3.1. Comparison of effective rate

**Figure 3.**
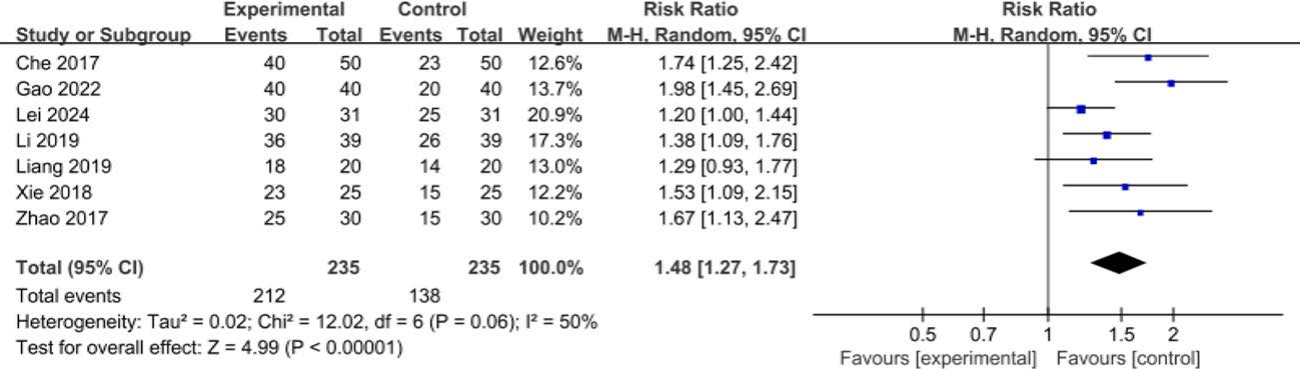
Meta-analysis of the comparison of effective rate between the 2 groups of patients. CI = confidence interval.

The 7 RCTs^[11–16,18]^ included a total of 470 patients (235 patients in the experimental group and 235 patients in the control group); the meta-analysis of the comparison of treatment efficiency used a random-effect model, and there was middle heterogeneity between the studies (*I*^2^ = 50%, *P* = .06); the complication rate was 90.21% (212/235) in the experimental group, and 58.72% (138/235) in the control group. The treatment efficiency of the experimental group was higher than that of the control group and the difference was statistically significant (RR = 1.48, 95% CI: [1.27–1.73]), as shown in Figure 3.

#### 3.3.2. *Comparison of pretreatment CD3^+^*

**Figure 4.**
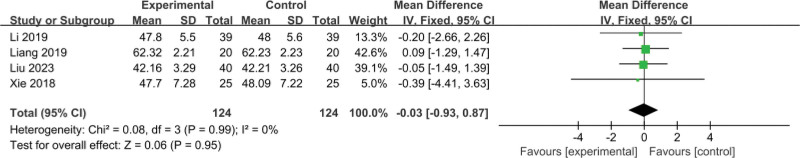
Meta-analysis of the comparison of pretreatment CD3^+^. CI = confidence interval, CD3^+^ = cluster of differentiation 3, SD = standard deviation.

The 4 RCTs^[11,13,14,17]^ included involved a total of 248 patients (124 patients in the experimental group and 124 patients in the control group); meta-analysis of the comparison of pretreatment CD3^+^, using a fixed-effect model, there was no heterogeneity between the studies (*I*^2^ = 0%, *P* = .99); the difference was not statistically significant (MD = −0.03, 95% CI: [−0.93 to 0.87, *P* = .95]), as shown in Figure 4.

#### 3.3.3. *Comparison of posttreatment CD3^+^*

**Figure 5.**
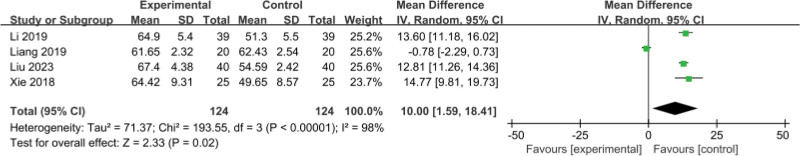
Meta-analysis of the comparison of posttreatment CD3^+^. CI = confidence interval, CD3^+^ = cluster of differentiation 3, SD = standard deviation.

The 4 RCTs^[11,13,14,17]^ included involved a total of 248 patients (124 patients in the experimental group and 124 patients in the control group); meta-analysis of the comparison of posttreatment CD3^+^, using a random-effect model, there was high heterogeneity between the studies (*I*^2^ = 98%, *P* < .001); the difference in the CD3^+^ of the patients of the 2 groups after treatment, was statistically significant [MD = 10.00, 95% CI: (1.59–18.41, *P* = .02)], as shown in Figure 5.

#### 3.3.4. *Comparison of pretreatment CD4^+^*

**Figure 6.**
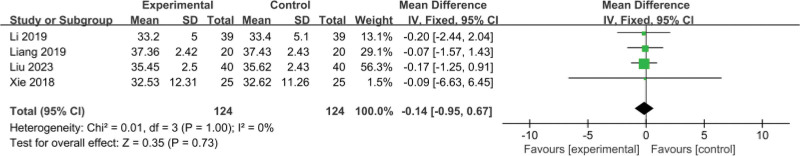
Meta-analysis of the comparison of pretreatment CD4^+^. CI = confidence interval, CD4^+^ = cluster of differentiation 4, SD = standard deviation.

The 4 RCTs^[11,13,14,17]^ included involved a total of 248 patients (124 patients in the experimental group and 124 cases in the control group); meta-analysis of the comparison of pretreatment CD4^+^ between the 2 groups, there was no heterogeneity between the studies (*I*^2^ = 0%, *P* = 1.00), and a fixed-effects model was used; there was no statistical significance in the level of pretreatment CD4^+^ in the 2 groups (MD = −0.14, 95% CI: [−0.95 to 0.67, *P* = .73]), as shown in Figure 6.

#### 3.3.5. *Comparison of posttreatment CD4^+^*

**Figure 7.**
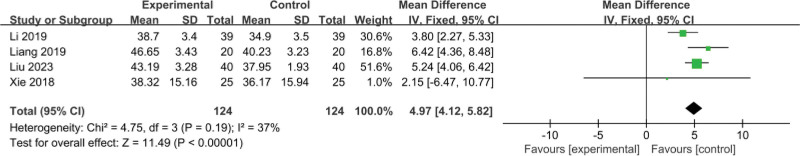
Meta-analysis of the comparison of posttreatment CD4^+^. CI = confidence interval, CD4^+^ = cluster of differentiation 4, SD = standard deviation.

The 4 RCTs^[11,13,14,17]^ included involved a total of 248 patients (124 patients in the experimental group and 124 cases in the control group); meta-analysis of the comparison of posttreatment CD4^+^ between the 2 groups, there was low heterogeneity between the studies (*I*^2^ = 37%, *P* = .19), and a fixed-effects model was used; there was a statistically significant posttreatment level of CD4^+^ in the 2 groups (MD = 4.97, 95% CI: [4.12–5.82, *P* < .001]), as shown in Figure 7.

#### 3.3.6. Comparison of pretreatment KPS

**Figure 8.**
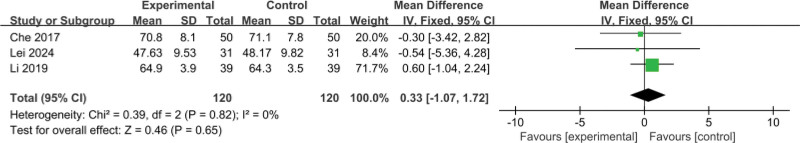
Meta-analysis of the comparison of pretreatment KPS. CI = confidence interval, KPS = Karnofsky performance status, SD = standard deviation.

The 3 included RCTs^[13,16,18]^ involved a total of 240 patients (120 patients in the experimental group and 120 patients in the control group); meta-analysis of the comparison of pretreatment KPS, using a fixed-effects model, showed no heterogeneity between studies (*I*^2^ = 0%, *P* = .82); the difference in the comparison of pretreatment KPS was not statistically significant (MD = 0.33, 95% CI: [−1.07 to 1.72, *P* = .65]), as shown in Figure 8.

#### 3.3.7. Comparison of posttreatment KPS

**Figure 9.**
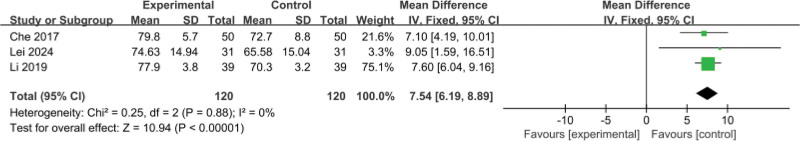
Meta-analysis of the comparison of posttreatment KPS. CI = confidence interval, KPS = Karnofsky performance status, SD = standard deviation.

The 3 RCTs^[13,16,18]^ included involved a total of 240 patients (120 patients in the experimental group and 120 patients in the control group); meta-analysis of the comparison of posttreatment KPS was performed with a fixed-effects model, showed no heterogeneity between studies (*I*^2^ = 0%, *P* = .88); and the difference between the comparison of posttreatment KPS was statistically significant (MD = 7.54, 95% CI: [6.19–8.89, *P* < .001]), as shown in Figure 9.

#### 3.3.8. Comparison of pretreatment FEV1

**Figure 10.**
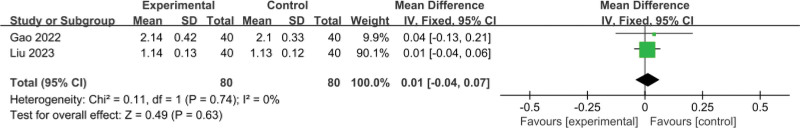
Meta-analysis of the comparison of pretreatment FEV1. CI = confidence interval, FEV1 = forced expiratory volume in one second, SD = standard deviation.

The 2 included RCTs^[15,17]^ involved a total of 160 patients (80 patients in the experimental group and 80 patients in the control group); meta-analysis of the comparison of pretreatment FEV1, using a fixed-effects model, showed no heterogeneity between studies (*I*^2^ = 0%, *P* = .74); the difference in the comparison of pretreatment FEV1 was not statistically significant (MD = 0.01, 95% CI: [−0.04 to 0.07, *P* = .63]), as shown in Figure 10.

#### 3.3.9. Comparison of posttreatment FEV1

**Figure 11.**
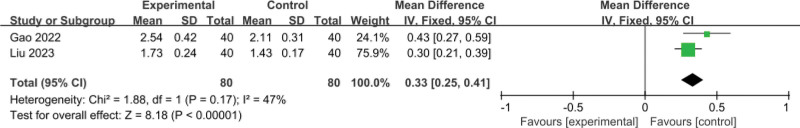
Meta-analysis of the comparison of posttreatment FEV1. CI = confidence interval, FEV1 = forced expiratory volume in one second, SD = standard deviation.

The 2 RCTs^[15,17]^ included involved a total of 160 patients (80 patients in the experimental group and 80 patients in the control group); meta-analysis of the comparison of posttreatment FEV1 was performed with a fixed-effects model, showed low heterogeneity between studies (*I*^2^ = 47%, *P* = .17); and the difference between the comparison of posttreatment FEV1 was statistically significant (MD = 0.33, 95% CI: [0.25–0.41, *P* < .001]), as shown in Figure 11.

### 3.4. Subgroup analysis and sensitivity analysis

#### 3.4.1. Subgroup analysis of effective rate

Some articles explicitly mentioned that the patients included in the study had a specific non-small-cell lung cancer (NSCLC), while 2 RCTs only mentioned lung cancer without explicitly stating it, so subgroups were divided in this way.

##### 3.4.1.1. Non-small cell lung cancer group

**Figure 12.**
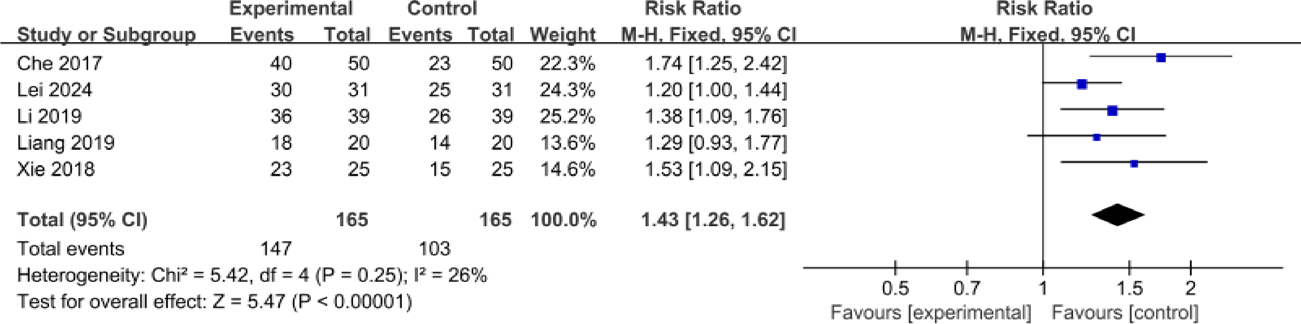
Subgroup analysis of effective rate after treatment in both groups of patients (non-small cell lung cancer group). CI = confidence interval.

The 5 RCTs^[11,13,14,16,18]^ included involved a total of 330 patients (165 cases in the test group and 165 cases in the control group); there was low heterogeneity between the studies (*I*^2^ = 26%, *P* = .25), and a fixed-effects model was used; there was statistical significance in the posttreatment effective rate in the 2 groups (MD = 1.43, 95% CI: [1.26–1.62, *P* < .001]), as shown in Figure 12.

##### 3.4.1.2. Lung cancer group

**Figure 13.**
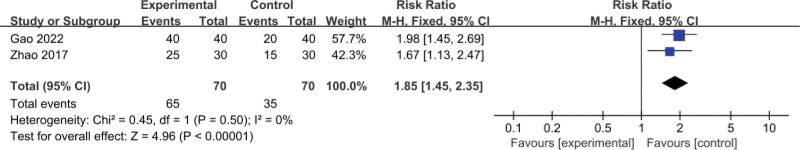
Subgroup analysis of effective rate after treatment in both groups of patients (lung cancer group). CI = confidence interval.

The 2 RCTs^[12,15]^ included involved a total of 140 patients (70 cases in the test group and 70 cases in the control group); there was no heterogeneity between the studies (*I*^2^ = 0%, *P* = .50), and a fixed-effects model was used; there was statistical significance in the posttreatment effective rate in the 2 groups (MD = 1.85, 95% CI: (1.45–2.35, *P* < .001]), as shown in Figure 13.

#### 3.4.2. *Sensitivity analysis of posttreatment CD3^+^*

**Figure 14.**
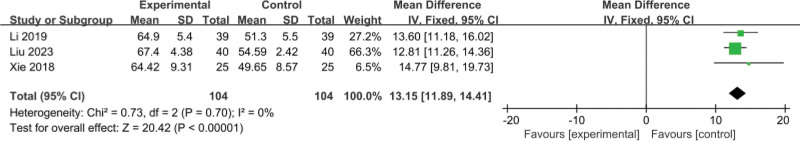
Meta-analysis of CD3 after treatment in both groups of patients (after subgroup analysis). CI = confidence interval, CD = cluster of differentiation, SD = standard deviation.

Data from 1 study that differed significantly from the other were also included in the analysis, considering that this study itself had a rigorous study design, but indicators with a high degree of heterogeneity. After carefully reading the article, it is inferred that this is related to the subtle adjustments in the drug composition. After excluding it, a sensitivity analysis was performed. The 3 RCTs^[13,14,17]^ included involved a total of 208 patients (104 cases in the test group and 104 cases in the control group); there was no heterogeneity between the studies (*I*^2^ = 0%, *P* = .70), and a fixed-effects model was used; there was statistical significance in the posttreatment CD3^+^ in the 2 groups (MD = 13.15, 95% CI: [11.89–14.41, *P* < .001]), as shown in Figure 14.

### 3.5. Analysis of publication bias based on effective rate outcomes

**Figure 15.**
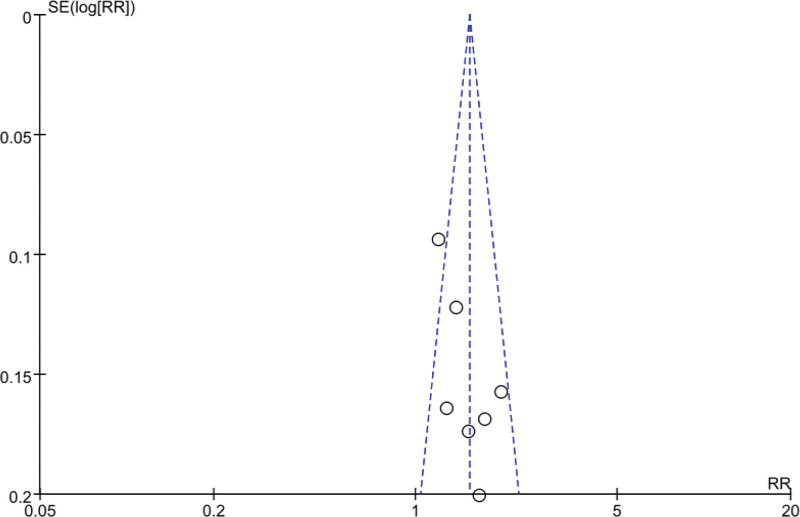
Funnel plot of publication bias test based on effective rate.

The distribution of the included studies was largely symmetrical on both sides of the funnel, suggesting that there was low probability of publication bias, as shown in Figure 15.

### 3.6. Comparison of evidence quality grading results

The quality of the literatures included in this study is all grade B. The literature quality is relatively high, and the risk bias is relatively small. However, only 1 study clearly mentions the allocation concealment, and the rest do not mention it. In future studies, it is necessary to further improve the design of allocation concealment. Among the GRADE evidence grading, 2 outcome indicators are of moderate quality, which improves the credibility of CD4^+^ and KPS. There are 3 outcome indicators of low quality, but the heterogeneity can be explained after sensitivity analysis. Researchers should standardize the experimental design and rigorously implement the process in future studies. As shown in Table 2.

**Table 2:** GRADE evidence grading evaluation form.

Outcomes	Number of studies	Evaluation of evidence quality					Sample size		Effect size	Evidence quality
		Risk of bias	Inconsistency	Indirectness	Imprecision	Other consideration	T	C		
Effective rate	7	−1*	−1†	0	0	0	235	235	RR = 1.48, 95% CI: (1.27–1.73)	Low
CD3^+^	4	−1*	−1†	0	0	0	124	124	MD = 10.00, 95% CI: (1.59–18.41)	Low
CD4^+^	4	−1*	0	0	0	0	124	124	MD = 4.97, 95% CI: (4.12–5.82)	Moderate
KPS	3	−1*	0	0	0	0	120	120	MD = 7.54, 95% CI: (6.19–8.89)	Moderate
FEV1	2	−1*	0	0	−1‡	0	80	80	MD = 0.33, 95% CI: (0.25–0.41)	Low

CD3^+^ = cluster of differentiation 3, CD4^+^ = cluster of differentiation 4, CI = confidence interval, FEV1 = forced expiratory volume in one second, KPS = Karnofsky performance status, MD = mean difference, RR = relative risk.

* Indicates that the included studies did not describe blinding or allocation concealment.

† Indicates heterogeneity >50%.

‡ Indicates small sample sizes of the included studies.

## 4. Discussion

A total of 8 RCTs with 550 lung cancer patients were included in this meta-analysis, and the results of meta-analysis showed that the improvement of the index levels after treatment in the experimental group were all significantly better than that in the control group. It indicates that the treatment of lung cancer patients with Erchen Decoction can promote recovery faster and possesses better overall efficacy. The meta-analysis of Erchen Decoction is mostly the enhancement and improvement of the original treatment, not the abandonment of the original treatment.

There was a high degree of heterogeneity in 1 indicator in this study, and after analysis, the reasons can be explained: although all patients have lung cancer, some have non-small cell carcinoma while others have small cell carcinoma. These 2 types of cancer have different etiologies and pathogenesis in TCM; because the patients’ conditions are not exactly the same, the specific composition of the formula will also be slightly adjusted.

There are certain limitations in this meta-analysis: the sample size of most RCTs is relatively small. In 1 RCT, there were only 20 patients in both the control and observation groups respectively, which will exert some influence on the assessment of the results; due to the limited number of RCTs, studies were included whenever ErChen Decoction was applied to treat Lung Cancer, without specifically differentiating between the types of lung cancer; and the absence of foreign RCTs on the application of ErChen Decoction means that the conclusions of this meta-analysis should be interpreted with caution and may benefit from additional research.

Lung cancer is a global health issue, with NSCLC accounting for 80% to 85% of cases.^[19]^ Most patients are diagnosed at an advanced stage, with a 5-year survival rate of only 15%.^[20]^ Small-cell lung cancer makes up approximately 15% of lung cancers, characterized by rapid proliferation, a strong tendency for early metastasis, poor prognosis, and a high correlation with exposure to tobacco carcinogens. Most patients have metastases at diagnosis, and only one-third are in the early stage, eligible for potentially curative multimodal therapy.^[21]^ According to TCM scholars, NSCLC progresses relatively slowly, and TCM often considers it closely related to “spleen deficiency and phlegm-dampness.” Especially in patients with lung adenocarcinoma, phlegm-dampness syndromes such as cough, excessive sputum, chest tightness, and white greasy tongue coating are common. Phlegm-dampness can also combine with blood stasis and heat-toxin, forming a pathological basis of “deficiency of the root and excess of the branch.” For NSCLC patients with phlegm-dampness as the main syndrome, Erchen Decoction can directly address the etiology and pathogenesis through drying dampness to resolve phlegm, invigorating the spleen, and regulating the stomach. For example, modifying Erchen Decoction for patients with spleen deficiency and phlegm-dampness (such as combining with Sijunzi Decoction to enhance spleen-invigorating power) may improve cough and expectoration symptoms, inhibit phlegm-dampness condensation in the tumor microenvironment, and align with the “chronic conditioning” treatment approach for NSCLC. Small cell lung cancer is highly malignant and progresses rapidly. TCM emphasizes “exuberant toxic pathogens,” with phlegm-dampness often existing as a “concomitant pathogen,” easily combining with heat-toxin and blood stasis-toxin to form a rapid pathogenesis of “phlegm-toxin intermingling.” Additionally, SCLC patients may experience transient phlegm-dampness obstruction due to spleen-stomach injury from radiotherapy and chemotherapy, but the core pathogenesis remains “toxic pathogens damaging vital qi,” with phlegm-dampness not being dominant. Using Erchen Decoction alone for SCLC may fail to curb toxic pathogen invasion due to the lack of “detoxifying and anti-cancer” effects.

## 5. Conclusion

The TCM concept of “holistic regulation” is important in lung cancer prevention and treatment, working by inhibiting inflammation, reducing oxidative stress, suppressing apoptosis and improving airway remodeling.^[22]^ Proper use of Erchen Decoction improves patients’ respiratory status and quality of life. Lung cancer patients have qi stagnation, phlegm coagulation and blood stasis alongside healthy qi deficiency, so treatment should tonify qi, resolve phlegm and activate blood circulation, without limiting to 1 or 2 medicines. Combining syndrome differentiation and disease identification is key to treatment.^[23]^

Under the current research conditions, Erchen Decoction shows certain potential in the adjuvant treatment of lung cancer. Nevertheless, further high-quality research is needed to verify its efficacy and safety to determine whether it is suitable for wide-scale promotion. When expanding the sample size, it should cover lung cancer patients from different regions and ethnic groups to enhance the representativeness of the sample.

## Author contributions

**Formal analysis:** Xueyan Lv.

**Funding acquisition:** Xiangni Zou.

**Validation:** Dongmei Wang.

**Writing – original draft:** Xiangni Zou.

**Writing – review & editing:** Xueyan Lv, Dongmei Wang.
